# Supplementary material for: Epigenetic control of topoisomerase 1 activity presents a cancer vulnerability
Source: Nat Commun. 2025 Aug 12;16:7458. doi: 10.1038/s41467-025-62598-w (PMC12343833; doi:10.1038/s41467-025-62598-w)
Supplement: Supplementary file 1 — Supplementary Information [file 41467_2025_62598_MOESM1_ESM.pdf]

SUPPLEMENTARY INFORMATION

Epigenetic control of Topoisomerase 1 activity presents a cancer vulnerability

Supplementary Figures

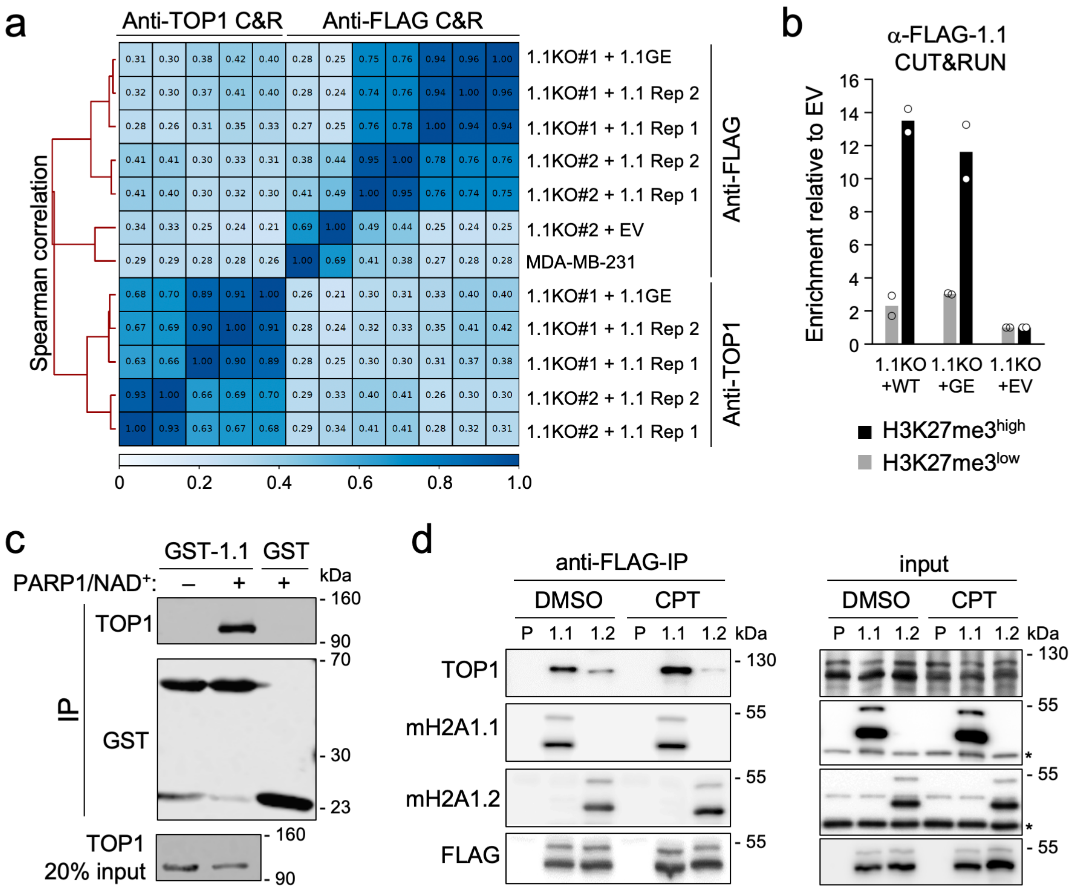

Supplementary Figure 1, related to Figure 1. a Unsupervised hierarchical clustering of the indicated CUT&RUN NGS samples based on Spearman Correlation Coefficients. 1.1KO#1 and 1.1KO#2 represent independent CRISPR knockout clones, Rep 1 and Rep 2 are independent replicates. **b** qPCR analysis of FLAG-macroH2A1.1 CUT&RUN samples from MDA-MB-231 macroH2A1.1 knockout (1.1KO) cells reconstituted with WT or G224E (GE) mutant macroH2A1.1, or empty vector (EV) at an H3K27me3^{high} heterochromatin region known to bind macroH2A1.1 and an H3K27me3^{low} control region [19], see Supplementary Table 1 for primer sequences. Samples were normalized to the EV control, n=2 independent replicates. **c** In vitro pull-down of full-length, recombinant, non-modified (–) or ADP-ribosylated (+) TOP1, using GST or a GST-tagged macroH2A1.1 macro-domain as bait. PARP1 (enzyme) and NAD^+ (substrate) were used for TOP1 ADP-ribosylation. A representative of two independent experiments is shown. **d** Western blot for the indicated proteins in nuclear lysates (input) or anti-FLAG IP samples from parental (P) 293 cells and FLAG-macroH2A1.1 (1.1) or FLAG-macroH2A1.2 (1.2) knock-in cells in the presence or absence of CPT treatment; * endogenous macroH2A1.1 or macroH2A1.2 protein. Source data for b, c and d are provided as a Source Data file.

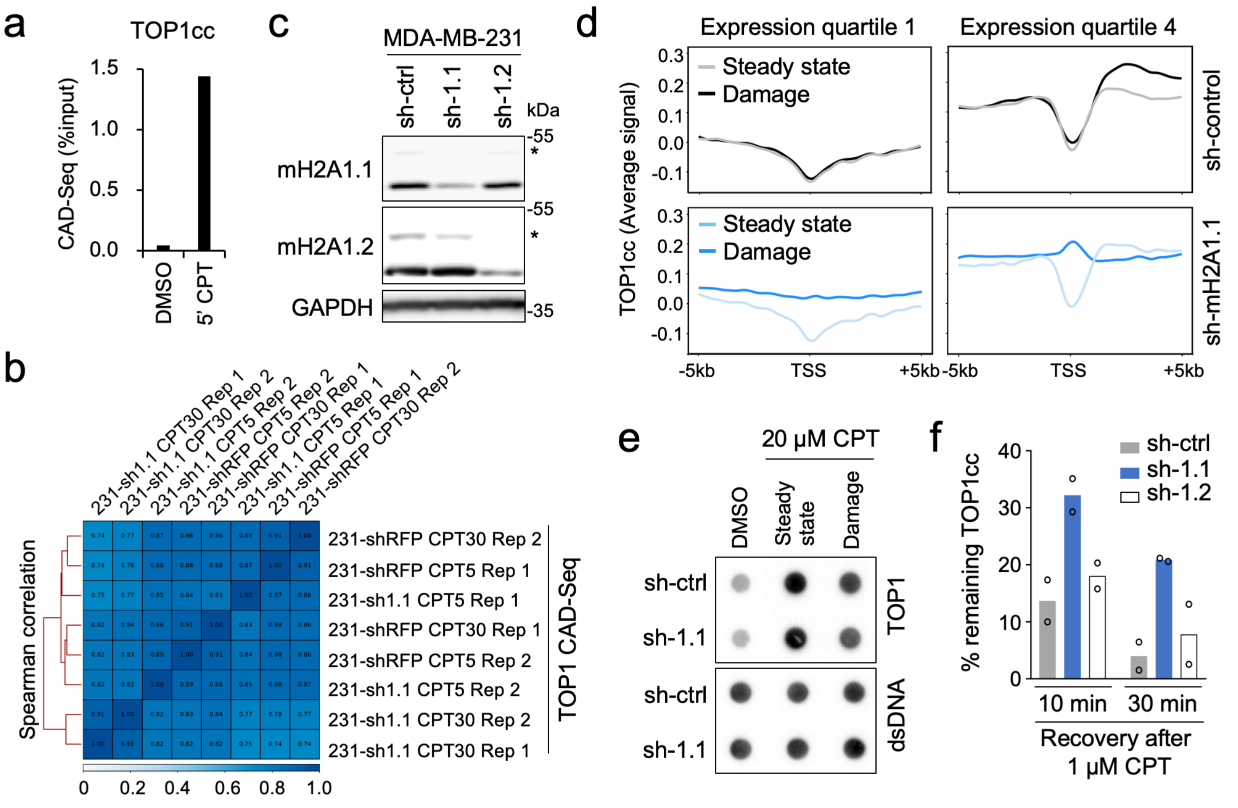

Supplementary Figure 2, related to Figure 2. a qPCR analysis of CAD-Seq IP DNA relative to input at the TOP1cc-rich MYC promoter as described in Ref. 30, in MDA-MB-231 cells in the absence (DMSO) or presence of TOP1cc stabilization (CPT 5'). **b** Unsupervised hierarchical clustering of the indicated TOP1 CAD-Seq samples as in Supplementary Figure 1a. CPT5 and CPT30: 5 min and 30 min CPT treatment, respectively; Rep: independent replicate experiment. **c** Western blot for the indicated proteins in MDA-MB-231 cells stably expressing shRNAs against macroH2A1.1 (sh-1.1), macroH2A1.2 (sh-1.2) or RFP (non-targeting control, sh-ctrl); * ubiquitinated macroH2A1 isoforms. A representative of three independent experiments is shown. **d** TOP1 CAD-Seq profiles for steady state (5' CPT) and damage conditions (30' CPT) as defined in Fig. 2c in cells expressing sh-RFP (sh-control) or sh-macroH2A1.1 (sh-mH2A1.1). CAD-Seq profiles were separated based on RNA-Seq derived gene expression quartiles, bottom (Q1) and top (Q4) quartiles are shown. **e** TOP1 RADAR assay with genomic DNA from MDA-MB-231 cells expressing the indicated shRNAs under TOP1 CAD-Seq treatment conditions as described in Fig. 2c; steady state: 5' CPT (20 µM), damage: 30' CPT (20 µM). A representative of two independent experiments is shown. **f** Quantification of TOP1cc turnover at the indicated time points after CPT treatment. The percent of remaining TOP1cc relative to 0' after CPT is shown for two independent RADAR assays (open circles), see Fig. 2f for a representative experiment. Source data for c, e and f are provided as a Source Data file.

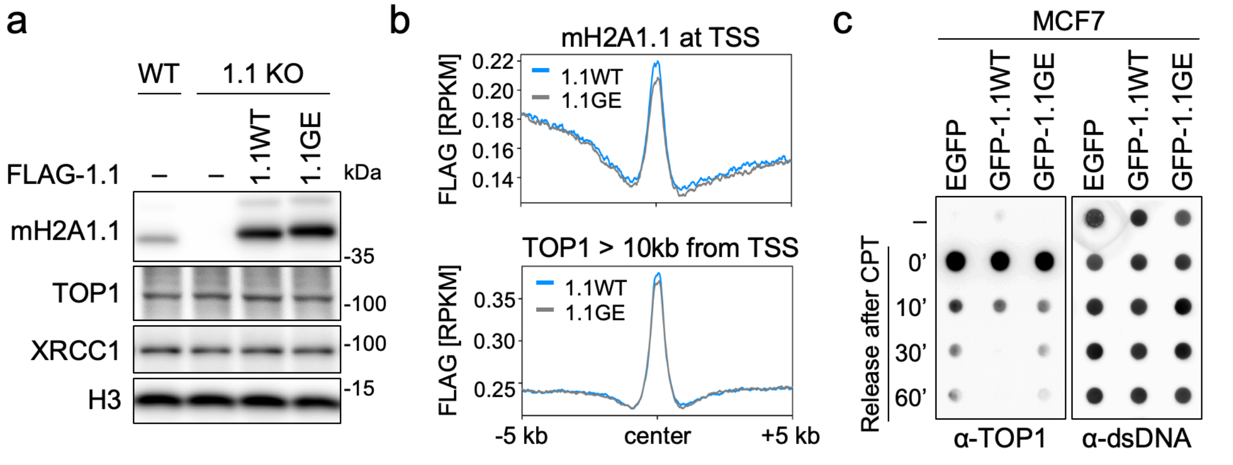

Supplementary Figure 3, related to Figure 3. a Western blot for the indicated proteins in MDA-MB-231 WT or macroH2A1.1 knockout (1.1 KO) cells, with or without stably integrated, FLAG-tagged wildtype (1.1WT) or G224E mutant macroH2A1.1 (1.1GE). A representative of two independent experiments is shown. **b** Profile plots for FLAG CUT&RUN signal in 1.1WT- or 1.1GE-expressing cells from (a). Signal was centered on TSS-proximal or TSS-distal TOP1 peaks. **c** RADAR assay as in Fig. 3a in MCF7 cells expressing GFP-tagged macroH2A1.1 (GFP-1.1) or macroH2A1.1 G224E (GFP-1.1GE), or an EGFP vector control. Similar results were obtained in MDA-MB-231 cells (see Figure 3). Source data for a and c are provided as a Source Data file.

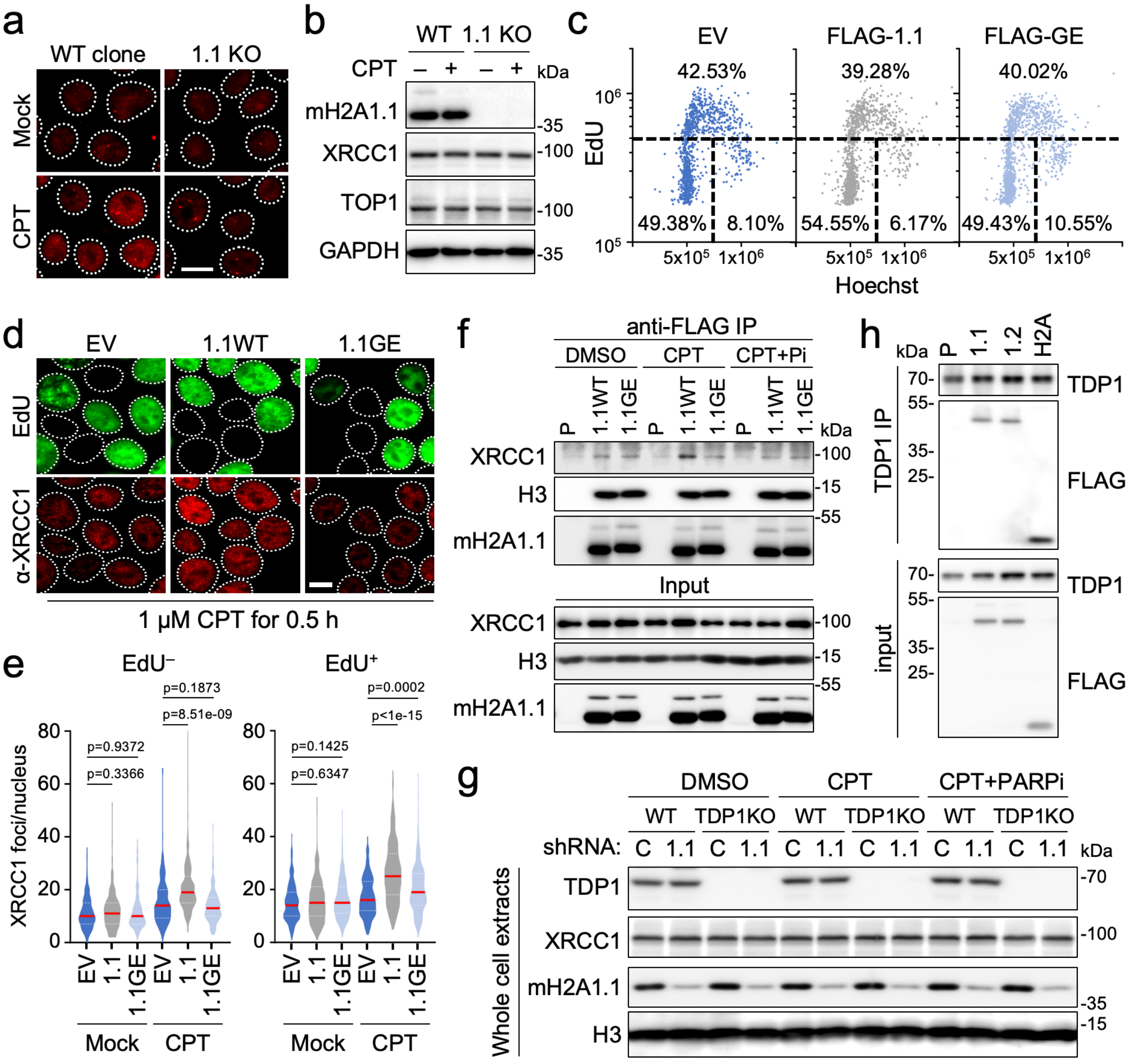

Supplementary Figure 4, related to Figure 4. a XRCC1 IF from MCF7 macroH2A1.1 knockout (1.1 KO) cells and a corresponding WT clone, quantified in Fig. 4b, similar results were obtained in a second, independent experiment. Scale bar: 10 µm. **b** Western blot for the indicated proteins in cells from (a) in the presence or absence of 1 µM CPT for 30 min. Similar results were obtained a second, independent experiment. **c** IF analysis of cell cycle profiles based on mean intensity distributions of DNA content (Hoechst) and EdU incorporation in MCF7 cell lines from Fig. 4d, labeled with EdU for 30 min, EV: empty vector. **d, e** XRCC1 foci quantification and representative images of cells in Fig. 4c separated based on EdU incorporation; red lines reflect the median, p values are based on two-sided Mann-Whitney U test for the indicated, pairwise comparisons. Scale bar: 10 µm. **f** Western blot for the indicated proteins in nuclear lysates (input) or IP lysates from parental 293 cells (P) and cells stably expressing wild-type (WT) or G224E mutant FLAG-macroH2A1.1 (1.1GE) in the presence or absence of CPT and PARP inhibitor (Pi). **g** Western blot for the indicated proteins in whole cell extracts from samples in Fig. 4e. **h** Western blot for the indicated proteins in nuclear lysates (input) or anti-TDP1 IP samples from parental (P) 293 cells and FLAG-macroH2A1.1 (1.1), FLAG-macroH2A1.2 (1.2) or FLAG-H2A (H2A) knock-in cells. Source data for b, c and e-h are provided as a Source Data file.

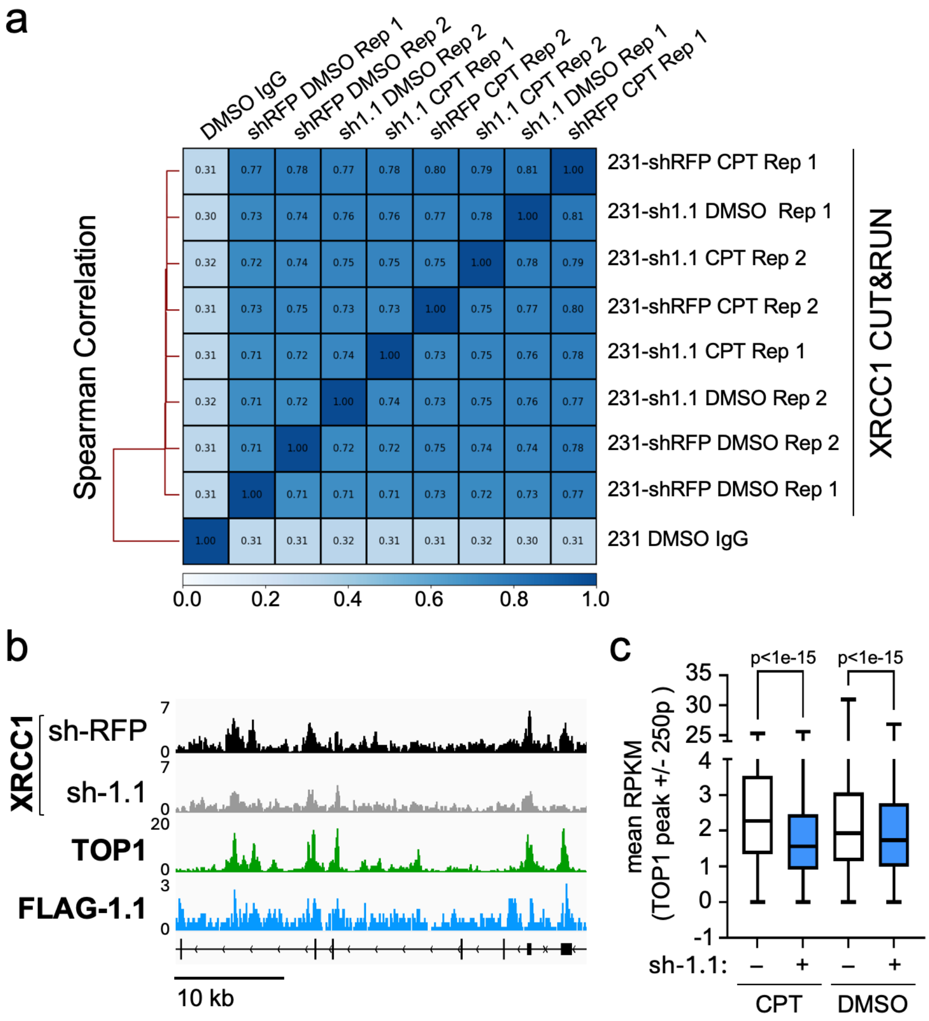

Supplementary Figure 5, related to Figure 4f, g. a Unsupervised hierarchical clustering of the indicated XRCC1 CUT&RUN NGS samples at TOP1 peaks. Cells were treated with DMSO or 1 µM CPT for 30'; Rep: independent replicate experiment. **b** IGV browser shots of averaged XRCC1 CUT&RUN replicate profiles from CPT-treated MDA-MD-231 cells expressing sh-RFP or sh-macroH2A1.1 (sh-1.1), genomic position: chr2:196,212,765-196,301,796. TOP1 and FLAG-macroH2A1.1 profiles from untreated MDA-MB-231 cells are shown as a reference (see Fig. 1b). **c** XRCC1 CUT&RUN signal within 500 bp of TOP1 peaks for indicated cell lines and treatment conditions, based on averaged RPKM profiles from two independent experiments. Box plots depict the distribution of average RPKM signal (TOP1 peak ± 250 bp) for the indicated knockdown and treatment conditions, each data point represents one TOP1 peak. Box limits represent upper and lower quartiles, whiskers minimum to maximum, and center lines the median; p values are based on two-sided Mann-Whitney U test for the indicated, pairwise comparisons.

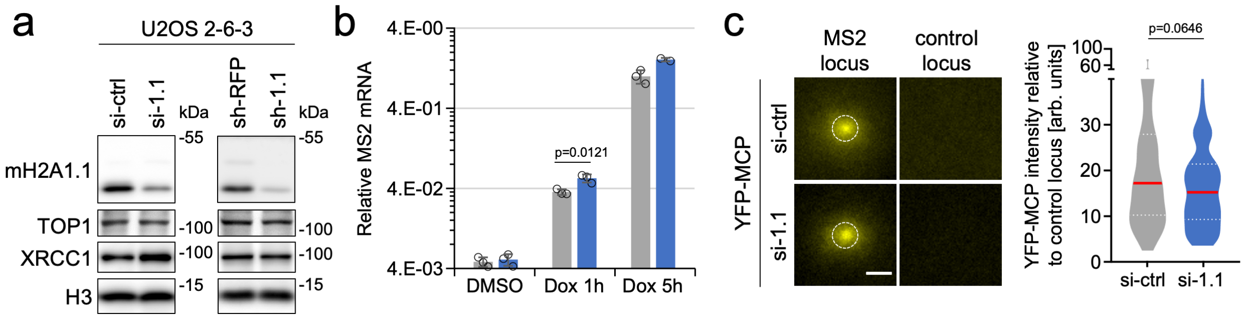

Supplementary Figure 6, related to Figure 5. a Western blot for the indicated proteins in U2OS MS2-reporter cells expressing siRNA against macroH2A1.1 (si-1.1) or a non-targeting control siRNA (si-ctrl). A representative of two independent experiments is shown. **b** RT-PCR for MS2 transcript in cells expressing si-1.1 or si-ctrl treated with DMSO or Dox for the indicated time points, PARGi was added for 30 min. mRNA levels were normalized to β-actin and rpl13a housekeeping genes. Bar graphs depict mean and SD, p values are based on two-sided Student's t-test (n=3 independent replicates, except si-1.1 5h Dox: n=2). **c** YFP-MCP intensity in cells from Fig. 5e. Cells were transfected with a non-targeting control siRNA (si-ctrl) or siRNA against macroH2A1.1 (si-1.1) and analyzed following 5 h Dox/PARGi treatment (n=82 MS2 foci). YFP-MCP intensities at the MS2 loci were normalized to the respective control loci, scale bar: 1 µm. Violin plots depict intensity distribution after background subtraction, red lines reflect the median, dashed lines depict quartile ranges, p value is based on two-sided Mann-Whitney U test. Similar results were obtained in two independent experiments. Source data are provided as a Source Data file.

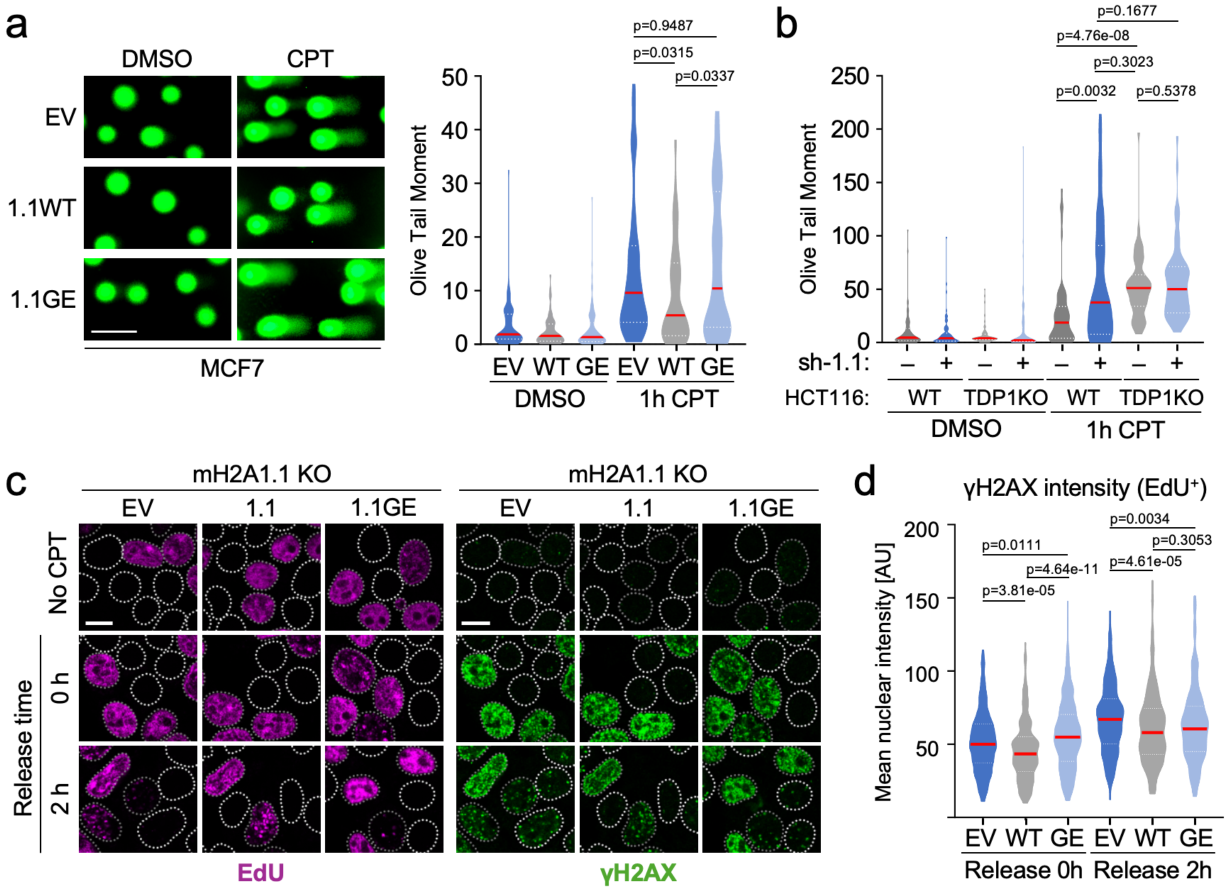

Supplementary Figure 7, related to Figure 6. a Alkaline comet assay in macroH2A1.1 KO (1.1 KO) MCF7 cells reconstituted with empty vector (EV), FLAG-macroH2A1.1 (1.1WT) or FLAG-macroH2A1.1 G224E (1.1GE). Cells were treated with 1 μM CPT or DMSO for 1 h, representative images are shown, scale bar: 100 μm; graphs depict Olive Tail Moment (n > 60 nuclei per sample, see source data for exact n). Similar results were obtained for WT and macroH2A1.1 KO cells, see Fig. 6a. **b** Alkaline comet assay in WT or TDP1 KO HCT116 cells expressing a control shRNA (–) or sh-macroH2A1.1 (+). Cells were treated as in (a), y axis depicts Olive Tail Moment (n > 50 nuclei per sample, see source data for exact n). Similar results were obtained for a second, independent replicate experiment. **c** EdU and γH2AX IF in 1.1 KO MCF7 cells reconstituted as in (a). Cells were treated with CPT (1 μM, 30 min) with or without 2 h release; no CPT samples were treated with DMSO. White nuclear outlines mark EdU^{-} cells, gray outlines EdU^{+} cells, scale bar: 10 μm. **d** Violin plots depicting γH2AX foci per nucleus in EdU^{+} cells from (c), n > 280 nuclei per sample, see source data for exact n. One of two independent experiments is shown. For all Violin plots, center lines (red) reflect the median, dotted lines upper and lower quartiles, and p values are based on two-sided Mann-Whitney U test for the indicated, pairwise comparisons. Source data for a, b and d are provided as a Source Data file.

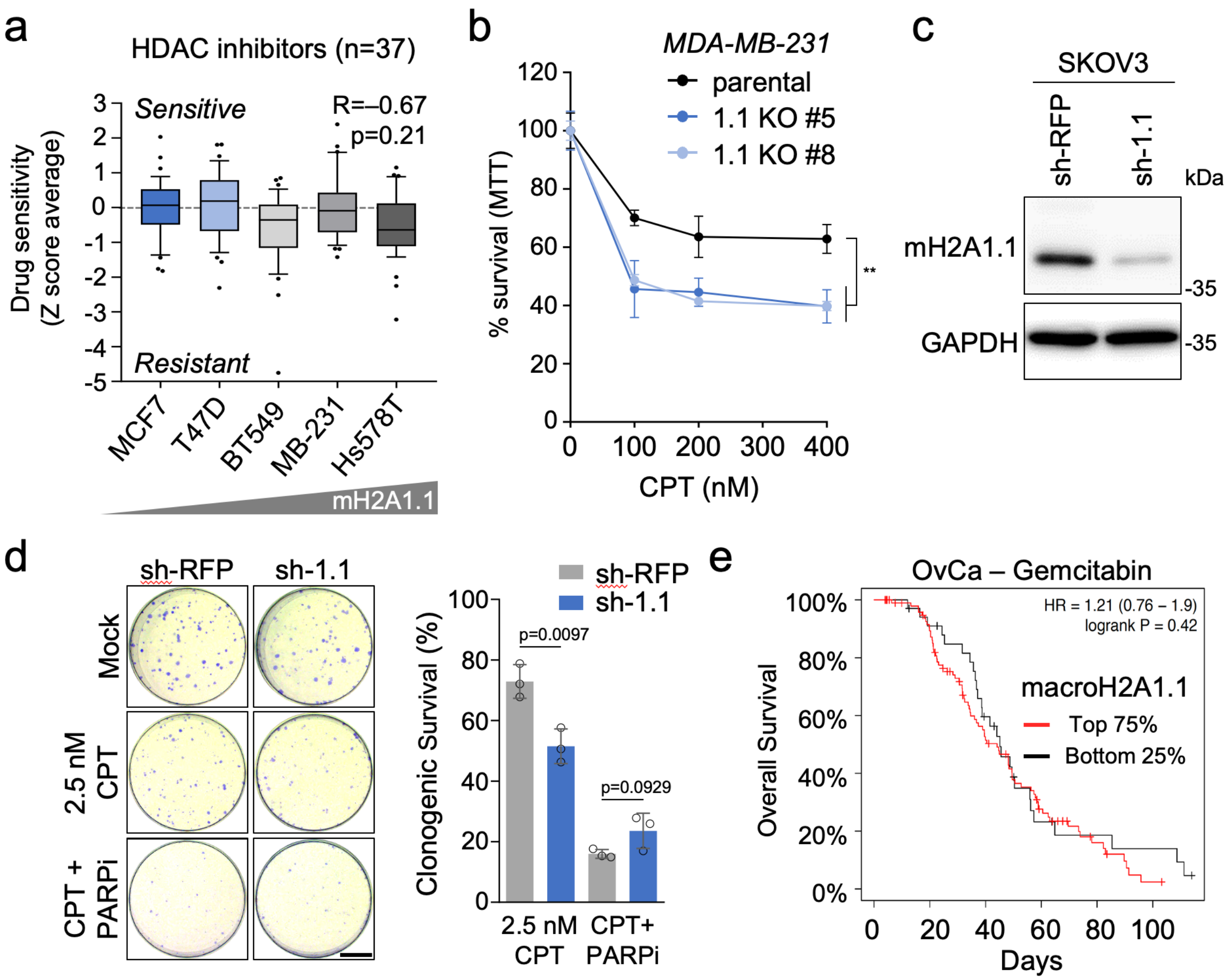

Supplementary Figure 8, related to Figure 7. a HDAC inhibitor activity levels based on drug screen in NCI60 breast cancer cell lines, as in Fig. 7b; n: number of compounds tested, p value is based on the two-sided Pearson's Correlation Coefficient of median drug activity scores. **b** Cell viability of parental MDA-MB-231 cells and two independent macroH2A1.1 KO (1.1KO) knockout clones in response to CPT treatment, measured as in Fig. 7c, data are presented as mean and SD (n=3 independent replicates). ** p=0.0061 for 1.1 KO #5, p=0.0016 for 1.1 KO #8, based on two-sided Student's t-test, relative to parental MDA-MB-231 cells. **c** Western blot for the indicated proteins in SKOV3 ovarian cancer cells stably expressing shRNAs against macroH2A1.1 or RFP (non-targeting control). **d** Clonogenic survival of cells from (c) in response to the indicated drug combinations, representative images are shown, scale bar: 1 cm. Survival was normalized to untreated cells for each shRNA. Data are presented as mean and SD (n=3 independent replicates). P values are based on two-sided Student's t-test. **e** Kaplan-Meyer analysis of overall survival of TCGA ovarian cancer patient subgroups where treatment regimens contained gemcitabine (n=135 patients). Patients were stratified by macroH2A1.1 mRNA expression as in Fig. 7h.

Supplementary Table 1: Primers and small RNA sequences.

Name	Sequence	Notes
**shRNA**		
sh-1.1	5’-CGACAAACACTGACTTCTAC-3’	
sh-1.2	5’-CTGAACCTTATTCACAGTGAA-3’	Ref. 53
sh-RFP	5'-CGTAATGCAGAAGAAGACCAT-3'	Ref. 53
**siRNA**		
si-1.1	5’-CGACAAACACUGACUUCUA-3’	Dharmacon
si-control	ON-TARGET Plus	Dharmacon
**guideRNA**		
E6b(1.1) KO 5’	5’-TTGGACCGAGACCCACGCAC-3’	1.1 exon-specific KO
E6b(1.1) KO 3’	5’ TTCTACATCGGTGGTGAAGT-3’	1.1 exon-specific KO
**Primers**		
E6b KO-detect F	5’- TGATCTCATGTGTGTGTTTCTCT-3’	1.1 KO screening
E6b KO-detect R	5’-TGGCTGACAGCTAGCTTTATT-3’	1.1 KO screening
$H3K27^{low}$ F	5’-CCTTTCACCCAGTACCTCATTT-3’	CUT&RUN validation
$H3K27^{low}$ R	5’-TCCTTCAATCCACCCATTCATC-3’	CUT&RUN validation
$H3K27^{high}$ F	5’-GGTGGCTGTAACTCTCTCGT-3’	CUT&RUN validation, Ref. 15
$H3K27^{high}$ R	5’-CCAGGCCCCAGATGATAGAG-3’	CUT&RUN validation, Ref. 21
RPL13A RT F	5’-GAAGTACCAGGCAGTGACAG-3’	RT-PCR
RPL13A RT R	5’-GGTCTTGAGGACCTCTGTG-3’	RT-PCR
b-Actin RT F	5’-TTCTACAATGAGCTGCGTGTGGCT-3’	RT-PCR
b-Actin RT R	5’-TCATCTTCTCGCGGTTGGCCT-3’	RT-PCR
MS2-reporter F	5’-TCATTAGATCCTGAGAACTTCA-3’	RT-PCR, Ref. 41
MS2-reporter R	5’-TTTTGGCAGAGGGAAAAAGA-3’	RT-PCR, Ref. 41
CMV-CFP (TSS) F	5’-TTTGACCTCCATAGAAGACACC-3’	CAD qPCR
CMV-CFP (TSS) R	5’-CGTCGCCGTCCAGCTCGACCAG-3’	CAD qPCR
CMV-Enh (upstr) F	5’-AGTAACGCCAATAGGGACTTTC-3’	CAD qPCR
CMV-Enh (upstr) R	5’-GGCGTACTTGGCATATGATACA-3’	CAD qPCR
