# Supplementary material for: Epigenetic control of topoisomerase 1 activity presents a cancer vulnerability
Source: Nat Commun. 2025 Aug 12;16:7458. doi: 10.1038/s41467-025-62598-w (PMC12343833; doi:10.1038/s41467-025-62598-w)
Supplement: Supplementary file 2 — Reporting Summary [file 41467_2025_62598_MOESM2_ESM.pdf]

nature portfolio

Corresponding author(s): Philipp Oberdoerffer

Last updated by author(s): Jul 7, 2025

Reporting Summary

Nature Portfolio wishes to improve the reproducibility of the work that we publish. This form provides structure for consistency and transparency in reporting. For further information on Nature Portfolio policies, see our Editorial Policies and the Editorial Policy Checklist.

Statistics

For all statistical analyses, confirm that the following items are present in the figure legend, table legend, main text, or Methods section.

n/a	Confirmed	
☐	☒	The exact sample size (*n*) for each experimental group/condition, given as a discrete number and unit of measurement
☐	☒	A statement on whether measurements were taken from distinct samples or whether the same sample was measured repeatedly
☐	☒	The statistical test(s) used AND whether they are one- or two-sided<br>*Only common tests should be described solely by name; describe more complex techniques in the Methods section.*
☒	☐	A description of all covariates tested
☒	☐	A description of any assumptions or corrections, such as tests of normality and adjustment for multiple comparisons
☐	☒	A full description of the statistical parameters including central tendency (e.g. means) or other basic estimates (e.g. regression coefficient) AND variation (e.g. standard deviation) or associated estimates of uncertainty (e.g. confidence intervals)
☐	☒	For null hypothesis testing, the test statistic (e.g. *F*, *t*, *r*) with confidence intervals, effect sizes, degrees of freedom and *P* value noted<br>*Give P values as exact values whenever suitable.*
☒	☐	For Bayesian analysis, information on the choice of priors and Markov chain Monte Carlo settings
☒	☐	For hierarchical and complex designs, identification of the appropriate level for tests and full reporting of outcomes
☐	☒	Estimates of effect sizes (e.g. Cohen's *d*, Pearson's *r*), indicating how they were calculated

Our web collection on statistics for biologists contains articles on many of the points above.

Software and code

Policy information about availability of computer code

Data collection	See data analysis
Data analysis	NGS QC: FastQC (v0.11.9); read trimming: cutadapt (v3.2); alignments: Bowtie2 (v2.4.1); read sorting and duplicate removal: Picard (v2.20.8), Samtools (v1.15.1); peak calling and analysis: SICER (v1.1), MACS2 (v2.2.9.1), deepTools (v3.5.5) bigwigAverage,multiBigwigSummary and plotCorrelation; annotations: ChIPseeker (v1.34.1); Jaccard index analysis: bedtools (v2.30); graphing and statistics: R (v4.2.1)

For manuscripts utilizing custom algorithms or software that are central to the research but not yet described in published literature, software must be made available to editors and reviewers. We strongly encourage code deposition in a community repository (e.g. GitHub). See the Nature Portfolio guidelines for submitting code & software for further information.

Data

Policy information about availability of data

All manuscripts must include a data availability statement. This statement should provide the following information, where applicable:

- Accession codes, unique identifiers, or web links for publicly available datasets
- A description of any restrictions on data availability
- For clinical datasets or third party data, please ensure that the statement adheres to our policy

The genomic data generated in this study have been deposited in the the database of Genotypes and Phenotypes (dbGaP) under accession code phs003729, https://www.ncbi.nlm.nih.gov/projects/gap/cgi-bin/study.cgi?study_id=phs003729.v2.p1. According to the to the NIH Genomic Data Sharing Policy, the large

human cell line-derived genomic data set generated in this study is subject to JHU Institutional Certification, which requires controlled data access. The genomic data are available for health/medical/biomedical research. To access the data, login to the dbGaP controlled-access portal to initiate a project. Source data are provided with this paper as indicated in the Figure legends.

Research involving human participants, their data, or biological material

Policy information about studies with human participants or human data. See also policy information about sex, gender (identity/presentation), and sexual orientation and race, ethnicity and racism.

Reporting on sex and gender	All human cell lines used in this study are commercially available. Human cell lines used for sequencing were MDA-MB231 breast cancer cell lines (ATCC HTB-26) derived from a female patient. All breast and ovarian cancer cell lines used for functional assays were derived from female patients. Gender was not considered as a variable for cell-based reporter assays.
Reporting on race, ethnicity, or other socially relevant groupings	N/A
Population characteristics	N/A
Recruitment	N/A
Ethics oversight	Johns Hopkins School of Medicine, Office of Human Subject Research

Note that full information on the approval of the study protocol must also be provided in the manuscript.

Field-specific reporting

Please select the one below that is the best fit for your research. If you are not sure, read the appropriate sections before making your selection.

☒ Life sciences ☐ Behavioural & social sciences ☐ Ecological, evolutionary & environmental sciences

For a reference copy of the document with all sections, see nature.com/documents/nr-reporting-summary-flat.pdf

Life sciences study design

All studies must disclose on these points even when the disclosure is negative.

Sample size	No specific method was used to determine sample size. A least three independent experiments were used where Student's t test analyses of replicates were performed. For image analysis, at least individual 50 data points were collected for each individual sample to allow for sufficient statistical power. High throughput sequencing was >14 Mio unique reads per sample. Statistical analyses were carried out using appropriate statistical tests, including Student’s t-test, Mann–Whitney U test, Spearman’s or Pearson rank correlation, using PRISM software or R studio. P values of less than 0.05 are considered statistically significant.
Data exclusions	Data points were only excluded in rare cases of obvious sample processing errors, such as loss of DNA, cell detachment during IF, verification of inefficient knockdown.
Replication	All experimentation is conducted with independent biological repeats, replicate numbers are indicated in the relevant Figure legends. At least two independent experiments were performed for NGS studies. Gene inactivation experiments were conducted using orthogonal approaches, including RNAi, CRISPR/Cas9 gene knockout and knockout with gene reconstitution. Whenever possible, key conclusions were verified by alternative means, e.g. two different types of cell survival assays, RADAR assay to validate TOP1-CAD-Seq, XRCC1 IF and chromatin fractionation, comparison of results across distinct cell lines.
Randomization	For NGS analysis of feature overlap, permuted Jaccard values were calculated based on random shuffles of both peak sets (n = 1,000 shuffles) using the shuffle command from bedtools within the bounds of the human genome assembly hg38. Due to small sample sizes of experimental and control conditions, and the well-defined nature of experimental perturbations, no randomization of sample groups was performed for biological assays.
Blinding	Key imaging analyses were performed double-blinded, colony formation counting was performed blind using automated colony counting

Reporting for specific materials, systems and methods

We require information from authors about some types of materials, experimental systems and methods used in many studies. Here, indicate whether each material, system or method listed is relevant to your study. If you are not sure if a list item applies to your research, read the appropriate section before selecting a response.

Materials & experimental systems

n/a	Involved in the study
☐	☒ Antibodies
☐	☒ Eukaryotic cell lines
☐	☐ Palaeontology and archaeology
☐	☐ Animals and other organisms
☐	☐ Clinical data
☐	☐ Dual use research of concern
☐	☐ Plants

Methods

n/a	Involved in the study
☐	☒ ChIP-seq
☐	☐ Flow cytometry
☐	☐ MRI-based neuroimaging

Antibodies

Antibodies used

α-macroH2A1.1: Cell Signaling Technologies, Cat#12455, RRID:AB_2797923
α-macroH2A1.2: Millipore, Cat#MABE61, RRID:AB_10807977
α-FLAG: Sigma-Aldrich, Cat#F1804, RRID:AB_262044
α-TOP1 (Mouse, for WB, RADAR): BD Biosciences, Cat#556597, RRID:AB_396474
α-TOP1 (Rabbit, for ChIP-seq, IF): Abcam, Cat#ab109374, RRID:AB_10861978
α-PARP1: Cell Signaling Technologies, Cat#9542, RRID:AB_2160739
α-GAPDH: Santa Cruz, Cat#sc-32233, RRID:AB_627679
α-H3: Cell Signaling Technologies, Cat#9715, RRID:AB_331563
α-XRCC1: Santa Cruz, Cat#sc-56254, RRID:AB_794191
α-XRCC1: Novus Biologicals, Cat# NBP1-87154, RRID: AB_11029388
α-TDP1: Santa Cruz, Cat# sc-365674, RRID:AB_10847225
α-TDP1: Bethyl, Cat# A301-618A, RRID:AB_1211359
α-gH2AX: Sigma-Aldrich, Cat# 05-636, RRID:AB_309864
α-GST: Abcam, Cat# ab9085, RRID: AB_306993
α-6X His tag® antibody [HIS.H8], Abcam, Cat# ab18184, RRID:AB_444306
α-dsDNA: Abcam, Cat#ab27156, RRID:AB_470907

Validation

Antibodies were obtained from commercial vendors. Antibody specificity was verified using immunological testing (western blotting, immunofluorescence, immunoprecipitation), including experimentation where the epitope or antigen is overexpressed (typically using ectopic expression vectors) and silenced (using RNAi or CRISPR knockout). We prioritize those antibodies with a solid record of use in multiple independent studies, and those which are extensively validated (e.g. by Human ProteinAtlas antibodies (Uhlen M. et al. Science 2015;347(6220):1260419).

Eukaryotic cell lines

Policy information about cell lines and Sex and Gender in Research

Cell line source(s)

MCF7 (ATCC HTB-22), human, female
MDA-MB-231 (ATCC HTB-26), human, female
MDA-MB-453, Hs-578T, T47D and BT-549 (from Daniele Gilkes lab, JHU), human, female
SKOV3 (from Tian-li Wang lab, JHU), human, female
HEK293 T-Rex, HEK293 (from Broad Institute, Cambridge), human, female
U2OS 2-6-3 cells (from Roger Greenberg), human, female
HCT116 TDP1 KO and WT cells (From Yves Pommier), human, male

Authentication

Cell lines were obtained from suppliers with long-standing, accredited repositories, authentication and distribution services (such as ATCC) whenever possible. Key cell lines obtained from collaborators have been subjected to authentication using short-tandem repeat (STR) analyses at the Johns Hopkins University Genetic Resources Core Facility (GRCF) prior to use in this study.

Mycoplasma contamination

Cells are regularly tested for the presence of mycoplasma using PCR-based detection methods. In addition, all new cell lines are tested for mycoplasma upon receipt. In rare cases where cells were found mycoplasma positive, they were discarded or treated with Plasmocin or Plasmocure (Invivogen) until mycoplasma was no longer detected. All data in this study were generated with or reproduced in mycoplasma negative cells.

Commonly misidentified lines
(See ICLAC register)

N/A

Palaeontology and Archaeology

Specimen provenance

Provide provenance information for specimens and describe permits that were obtained for the work (including the name of the issuing authority, the date of issue, and any identifying information). Permits should encompass collection and, where applicable, export.

Specimen deposition	*Indicate where the specimens have been deposited to permit free access by other researchers.*
Dating methods	*If new dates are provided, describe how they were obtained (e.g. collection, storage, sample pretreatment and measurement), where they were obtained (i.e. lab name), the calibration program and the protocol for quality assurance OR state that no new dates are provided.*

☐ Tick this box to confirm that the raw and calibrated dates are available in the paper or in Supplementary Information.

Ethics oversight	*Identify the organization(s) that approved or provided guidance on the study protocol, OR state that no ethical approval or guidance was required and explain why not.*

Note that full information on the approval of the study protocol must also be provided in the manuscript.

Animals and other research organisms

Policy information about studies involving animals; ARRIVE guidelines recommended for reporting animal research, and Sex and Gender in Research

Laboratory animals	*For laboratory animals, report species, strain and age OR state that the study did not involve laboratory animals.*
Wild animals	*Provide details on animals observed in or captured in the field; report species and age where possible. Describe how animals were caught and transported and what happened to captive animals after the study (if killed, explain why and describe method; if released, say where and when) OR state that the study did not involve wild animals.*
Reporting on sex	*Indicate if findings apply to only one sex; describe whether sex was considered in study design, methods used for assigning sex. Provide data disaggregated for sex where this information has been collected in the source data as appropriate; provide overall numbers in this Reporting Summary. Please state if this information has not been collected. Report sex-based analyses where performed, justify reasons for lack of sex-based analysis.*
Field-collected samples	*For laboratory work with field-collected samples, describe all relevant parameters such as housing, maintenance, temperature, photoperiod and end-of-experiment protocol OR state that the study did not involve samples collected from the field.*
Ethics oversight	*Identify the organization(s) that approved or provided guidance on the study protocol, OR state that no ethical approval or guidance was required and explain why not.*

Note that full information on the approval of the study protocol must also be provided in the manuscript.

Clinical data

Policy information about clinical studies
All manuscripts should comply with the ICMJE guidelines for publication of clinical research and a completed CONSORT checklist must be included with all submissions.

Clinical trial registration	*Provide the trial registration number from ClinicalTrials.gov or an equivalent agency.*
Study protocol	*Note where the full trial protocol can be accessed OR if not available, explain why.*
Data collection	*Describe the settings and locales of data collection, noting the time periods of recruitment and data collection.*
Outcomes	*Describe how you pre-defined primary and secondary outcome measures and how you assessed these measures.*

Dual use research of concern

Policy information about dual use research of concern

Hazards

Could the accidental, deliberate or reckless misuse of agents or technologies generated in the work, or the application of information presented in the manuscript, pose a threat to:

No	Yes	
☒	☐	Public health
☒	☐	National security
☒	☐	Crops and/or livestock
☒	☐	Ecosystems
☒	☐	Any other significant area

Experiments of concern

Does the work involve any of these experiments of concern:

No	Yes	
☒	☐	Demonstrate how to render a vaccine ineffective
☒	☐	Confer resistance to therapeutically useful antibiotics or antiviral agents
☒	☐	Enhance the virulence of a pathogen or render a nonpathogen virulent
☒	☐	Increase transmissibility of a pathogen
☒	☐	Alter the host range of a pathogen
☒	☐	Enable evasion of diagnostic/detection modalities
☒	☐	Enable the weaponization of a biological agent or toxin
☒	☐	Any other potentially harmful combination of experiments and agents

Plants

Seed stocks

Report on the source of all seed stocks or other plant material used. If applicable, state the seed stock centre and catalogue number. If plant specimens were collected from the field, describe the collection location, date and sampling procedures.

Novel plant genotypes

Describe the methods by which all novel plant genotypes were produced. This includes those generated by transgenic approaches, gene editing, chemical/radiation-based mutagenesis and hybridization. For transgenic lines, describe the transformation method, the number of independent lines analyzed and the generation upon which experiments were performed. For gene-edited lines, describe the editor used, the endogenous sequence targeted for editing, the targeting guide RNA sequence (if applicable) and how the editor was applied.

Authentication

Describe any authentication procedures for each seed stock used or novel genotype generated. Describe any experiments used to assess the effect of a mutation and, where applicable, how potential secondary effects (e.g. second site T-DNA insertions, mosiacism, off-target gene editing) were examined.

ChIP-seq

Data deposition

☒ Confirm that both raw and final processed data have been deposited in a public database such as GEO.

☐ Confirm that you have deposited or provided access to graph files (e.g. BED files) for the called peaks.

Data access links
May remain private before publication.

For "Initial submission" or "Revised version" documents, provide reviewer access links. For your "Final submission" document, provide a link to the deposited data.

Files in database submission

231_FLAG-CR.bam
231-KO1_F-mH2A1_FLAG-CR1.bam
231-KO1_F-mH2A1_FLAG-CR2.bam
231-KO1_F-mH2A1_TOP1-CR1.bam
231-KO1_F-mH2A1_TOP1-CR2.bam
231-KO1_F-mH2A1GE_FLAG-CR.bam
231-KO1_F-mH2A1GE_TOP1-CR.bam
231-KO2_EV_FLAG-CR.bam
231-KO2_F-mH2A1_FLAG-CR1.bam
231-KO2_F-mH2A1_FLAG-CR2.bam
231-KO2_F-mH2A1_TOP1-CR1.bam
231-KO2_F-mH2A1_TOP1-CR2.bam
231_sh-RFP_XRCC1_CPT_CR1.bam
231_sh-RFP_XRCC1_CPT_CR2.bam
231_sh-RFP_XRCC1_D_CR1.bam
231_sh-RFP_XRCC1_D_CR2.bam
231_sh-mH2A1_XRCC1_CPT_CR1.bam
231_sh-mH2A1_XRCC1_CPT_CR2.bam
231_sh-mH2A1_XRCC1_D_CR1.bam
231_sh-mH2A1_XRCC1_D_CR2.bam
231_IgG_CR.bam
231_sh-mH2A1_CPT30_CAD1.bam
231_sh-mH2A1_CPT30_CAD2.bam
231_sh-mH2A1_CPT5_CAD1.bam
231_sh-mH2A1_CPT5_CAD2.bam
231_sh-RFP_CPT30_CAD1.bam
231_sh-RFP_CPT30_CAD2.bam
231_sh-RFP_CPT5_CAD1.bam
231_sh-RFP_CPT5_CAD2.bam

231_CPT5_input_CAD.bam
231_CPT5_CAD.bam

Genome browser session
(e.g. UCSC)

Provide a link to an anonymized genome browser session for "Initial submission" and "Revised version" documents only, to enable peer review. Write "no longer applicable" for "Final submission" documents.

Methodology

Replicates

With exception of mH2A1GE, parental MDA-MB-231 CAD seq (231CPT5_CAD) and negative controls (231_FLAG_CR, 231_KO2_EV_FLAG-CR), all analyses were performed in biological replicates (labeled "1" and "2"). For some Cut&RUN (CR) samples, two independent knockout clones ("KO1" and "KO2") were used, reconstituted with FLAG-tagged macroH2A1.1 or GE mutation ("F-mH2A1" or F-mH2A1GE).

Sequencing depth

231_FLAG-CR.bam: 14285642 unique reads; PE
231-KO1_F-mH2A1_FLAG-CR1.bam: 20332062 unique reads; PE
231-KO1_F-mH2A1_FLAG-CR2.bam: 22093951 unique reads; PE
231-KO1_F-mH2A1_TOP1-CR1.bam: 30110235 unique reads; PE
231-KO1_F-mH2A1_TOP1-CR2.bam: 16290303 unique reads; PE
231-KO1_F-mH2A1GE_FLAG-CR.bam: 21929321 unique reads; PE
231-KO1_F-mH2A1GE_TOP1-CR.bam: 16546684 unique reads; PE
231-KO2_EV_FLAG-CR.bam: 20771172 unique reads; PE
231-KO2_F-mH2A1_FLAG-CR1.bam: 23572103 unique reads; PE
231-KO2_F-mH2A1_FLAG-CR2.bam: 22906230 unique reads; PE
231-KO2_F-mH2A1_TOP1-CR1.bam: 20934441 unique reads; PE
231-KO2_F-mH2A1_TOP1-CR2.bam: 22151021 unique reads; PE
231_sh-RFP_XRCC1_CPT_CR1.bam: 24263491 unique reads; PE
231_sh-RFP_XRCC1_CPT_CR2.bam: 20055411 unique reads; PE
231_sh-RFP_XRCC1_D_CR1.bam: 14517036 unique reads; PE
231_sh-RFP_XRCC1_D_CR2.bam: 15515553 unique reads; PE
231_sh-mH2A1_XRCC1_CPT_CR1.bam: 16753628 unique reads; PE
231_sh-mH2A1_XRCC1_CPT_CR2.bam: 18020301 unique reads; PE
231_sh-mH2A1_XRCC1_D_CR1.bam: 17118926 unique reads; PE
231_sh-mH2A1_XRCC1_D_CR2.bam: 15663353 unique reads; PE
231_IgG_CR.bam: 22807665 unique reads; PE
231_sh-mH2A1_CPT30_CAD1.bam: 30839199 unique reads; PE
231_sh-mH2A1_CPT30_CAD2.bam: 34166733 unique reads; PE
231_sh-mH2A1_CPT5_CAD1.bam: 14586951 unique reads; PE
231_sh-mH2A1_CPT5_CAD2.bam: 20584206 unique reads; PE
231_sh-RFP_CPT30_CAD1.bam: 29872290 unique reads; PE
231_sh-RFP_CPT30_CAD2.bam: 23316907 unique reads; PE
231_sh-RFP_CPT5_CAD1.bam: 28674992 unique reads; PE
231_sh-RFP_CPT5_CAD2.bam: 26997396 unique reads; PE
231_CPT5_input_CAD.bam: 20702669 unique reads; SE
231_CPT5_CAD.bam: 16522100 unique reads; SE

Antibodies

α-TOP1 (Rabbit, for ChIP-seq, IF): Abcam, Cat#ab109374, RRID:AB_10861978
α-FLAG: Sigma-Aldrich, Cat#F1804, RRID:AB_262044
α-XRCC1: Novus Biologicals, Cat# NBP1-87154, RRID: AB_11029388

Peak calling parameters

FLAG-macroH2A1.1 broad peaks were called using SICER with 200 bp window size, 150 fragment size, and 600 bp gap size, and default FDR <0.01. TOP1 narrow peaks were called using MACS2 and default FDR < 0.05.

Data quality

The quality of the raw sequenced reads was confirmed using FastQC.

Software

See data analysis above

Flow Cytometry

Plots

Confirm that:

☐ The axis labels state the marker and fluorochrome used (e.g. CD4-FITC).

☐ The axis scales are clearly visible. Include numbers along axes only for bottom left plot of group (a 'group' is an analysis of identical markers).

☐ All plots are contour plots with outliers or pseudocolor plots.

☐ A numerical value for number of cells or percentage (with statistics) is provided.

Methodology

Sample preparation

Describe the sample preparation, detailing the biological source of the cells and any tissue processing steps used.

Instrument	*Identify the instrument used for data collection, specifying make and model number.*
Software	*Describe the software used to collect and analyze the flow cytometry data. For custom code that has been deposited into a community repository, provide accession details.*
Cell population abundance	*Describe the abundance of the relevant cell populations within post-sort fractions, providing details on the purity of the samples and how it was determined.*
Gating strategy	*Describe the gating strategy used for all relevant experiments, specifying the preliminary FSC/SSC gates of the starting cell population, indicating where boundaries between "positive" and "negative" staining cell populations are defined.*

☐ Tick this box to confirm that a figure exemplifying the gating strategy is provided in the Supplementary Information.

Magnetic resonance imaging

Experimental design

Design type	*Indicate task or resting state; event-related or block design.*
Design specifications	*Specify the number of blocks, trials or experimental units per session and/or subject, and specify the length of each trial or block (if trials are blocked) and interval between trials.*
Behavioral performance measures	*State number and/or type of variables recorded (e.g. correct button press, response time) and what statistics were used to establish that the subjects were performing the task as expected (e.g. mean, range, and/or standard deviation across subjects).*

Acquisition

Imaging type(s)	*Specify: functional, structural, diffusion, perfusion.*
Field strength	*Specify in Tesla*
Sequence & imaging parameters	*Specify the pulse sequence type (gradient echo, spin echo, etc.), imaging type (EPI, spiral, etc.), field of view, matrix size, slice thickness, orientation and TE/TR/flip angle.*
Area of acquisition	*State whether a whole brain scan was used OR define the area of acquisition, describing how the region was determined.*

Diffusion MRI ☐ Used ☐ Not used

Preprocessing

Preprocessing software	*Provide detail on software version and revision number and on specific parameters (model/functions, brain extraction, segmentation, smoothing kernel size, etc.).*
Normalization	*If data were normalized/standardized, describe the approach(es): specify linear or non-linear and define image types used for transformation OR indicate that data were not normalized and explain rationale for lack of normalization.*
Normalization template	*Describe the template used for normalization/transformation, specifying subject space or group standardized space (e.g. original Talairach, MNI305, ICBM152) OR indicate that the data were not normalized.*
Noise and artifact removal	*Describe your procedure(s) for artifact and structured noise removal, specifying motion parameters, tissue signals and physiological signals (heart rate, respiration).*
Volume censoring	*Define your software and/or method and criteria for volume censoring, and state the extent of such censoring.*

Statistical modeling & inference

Model type and settings	*Specify type (mass univariate, multivariate, RSA, predictive, etc.) and describe essential details of the model at the first and second levels (e.g. fixed, random or mixed effects; drift or auto-correlation).*
Effect(s) tested	*Define precise effect in terms of the task or stimulus conditions instead of psychological concepts and indicate whether ANOVA or factorial designs were used.*

Specify type of analysis: ☐ Whole brain ☐ ROI-based ☐ Both

Statistic type for inference (See Eklund et al. 2016)	*Specify voxel-wise or cluster-wise and report all relevant parameters for cluster-wise methods.*
Correction	*Describe the type of correction and how it is obtained for multiple comparisons (e.g. FWE, FDR, permutation or Monte Carlo).*

Models & analysis

n/a	Involved in the study
☐	☐ Functional and/or effective connectivity
☐	☐ Graph analysis
☐	☐ Multivariate modeling or predictive analysis

Functional and/or effective connectivity	*Report the measures of dependence used and the model details (e.g. Pearson correlation, partial correlation, mutual information).*
Graph analysis	*Report the dependent variable and connectivity measure, specifying weighted graph or binarized graph, subject- or group-level, and the global and/or node summaries used (e.g. clustering coefficient, efficiency, etc.).*
Multivariate modeling and predictive analysis	*Specify independent variables, features extraction and dimension reduction, model, training and evaluation metrics.*
